# Identification of a CircRNA-miRNA-mRNA Network and Integrated Analysis of Immune Infiltration in Oral Squamous Cell Carcinoma

**DOI:** 10.7150/jca.79967

**Published:** 2023-01-01

**Authors:** Dehua Hu, Yufeng Cai, Lan He, Hao Jiang

**Affiliations:** 1 Department of Biomedical Informatics, School of Life Sciences, Central South University, Changsha, Hunan 410078, China.; 2 College of Biology, Hunan University, Changsha, Hunan 410078, China.

**Keywords:** Oral squamous cell carcinoma, circRNA, ceRNA, immune infiltration, bioinformatics

## Abstract

Oral squamous cell carcinoma (OSCC) is a highly invasive type of head and neck cancer. Circular RNA (circRNA) acts as a competing endogenous RNA (ceRNA) and involves in pathogenesis of many diseases. However, the circRNA-miRNA-mRNA network and relationship between ceRNA and immune infiltration in OSCC remain unknown. In this study, we established a ceRNA network, including 89 circRNAs, 43 miRNAs and 223 mRNAs, and found that 233 genes are mainly related to malignant signalling pathways (including "Integrin family cell surface interactions" and "Epithelial-to-mesenchymal transition" pathways) and five potential biomarkers (SLC20A1, PITX2, hsa-mir-135b, hsa-mir-377 and hsa-let-7c). Meanwhile, we established a prognostic model based on clinical risk, and revealed the relationship between immune infiltrating cells and biomarkers in OSCC. Taken together, our study is helpful to reveal the pathogenesis of oral squamous cell carcinoma.

## 1. Introduction

Oral squamous cell carcinoma (OSCC) is a common and highly invasive type of head and neck cancer that accounts for more than 90% of oral cancers 1, 2. According to GLOBOCAN statistics, there were an estimated 377,713 new cases of cancers of the lip and oral cavity and 177,757 associated deaths worldwide in 2020 3. The incidence of OSCC is increasing year by year; this is attributed to risk factors, including smoking, drinking, and human papillomavirus infection 1. OSCC is prone to metastasis and local recurrence, and the efficacy of conventional treatment with surgery, radiotherapy, and chemotherapy is unsatisfactory 4. Targeted therapies aimed at sites including programmed cell death protein-1 (PD-1) and EGFR have shown good potential in clinical practice 5. The choice of treatment for OSCC depends on the stage of the tumour. However, the commonly used TNM staging system is insufficient to evaluate the prognosis of OSCC patients 6. Previous bioinformatics analyses have explored the long noncoding RNA (lncRNA)-related ceRNA network in OSCC 7-9. A potential role of lncRNAs as biomarkers in head and neck squamous cell carcinoma has also been reported 10. However, no circRNA-related ceRNA network has been established, although this may provide more stable biomarkers. The relationship between the ceRNA network and immune infiltration in OSCC has not yet been reported. Exploring the internal mechanism of OSCC and the role of immune infiltration is critical to the further development of such immunotherapies. Circular RNAs (circRNAs), which are closed-ring molecules formed by reverse splicing and covalent association between their 3' and 5' ends 11, represent a recently rediscovered class of covalently closed transcripts 12. MicroRNAs (miRNAs) are noncoding RNAs with lengths <22 nucleotides that complementally bind to mRNA to inhibit gene expression 13. Although circRNAs can also interact with proteins, translate themselves, or act on other ribonucleic acids, their most widely studied function is their ability to act as competing endogenous RNAs (ceRNAs), serving as miRNA "sponges" to regulate gene expression through a circRNA-miRNA-mRNA network 14.

CircRNAs participate in cell proliferation, invasion, migration, and immunity in tumours 15. Studies have revealed various roles of circRNAs in OSCC. For example, circRNA_100290 antagonizes miR-378a-induced GLUT1 inhibition, thereby promoting glycolysis and cell proliferation in OSCC 16. Hsa_circ_0001162 blocks miR-149 to improve the stability of MMP9 mRNA, thereby promoting the metastasis of OSCC 17. Hsa_circ_0055538 acts on the p53/Bcl-2/caspase signalling pathway to inhibit tumour formation, playing a protective role in OSCC 18. CircRNAs are ideal diagnostic and prognostic biomarkers owing to their stability and tumour specificity 10. However, there has been no systematic analysis of the ceRNA network in OSCC. Immune cell infiltration in the tumour microenvironment may affect the occurrence, development, invasion, and prognosis of tumours 19, yet the relationship between the ceRNA network and immune infiltration in OSCC has not yet been reported.

In this study, we screened differentially expressed circRNAs (DEcircRNAs), differentially expressed miRNAs (DEmiRNAs), and differentially expressed mRNAs (DEmRNAs) between OSCC and normal tissues by bioinformatics methods, constructed a circRNA-miRNA-mRNA-related ceRNA network in OSCC, and evaluated the biological pathways associated with the mRNAs. We screened five biomarkers and established a prognostic model. In addition, we evaluated immune infiltration in OSCC and comprehensively analysed the correlations between immune cells and biomarkers. We constructed a systemic ceRNA network for OSCC and further discussed its relationship with immune infiltration. These results are expected to provide biomarkers of prognosis and indicate potential novel strategies for the treatment of OSCC.

## 2. Materials and methods

### 2.1 Differential expression and network analyses of raw data

The National Center for Biotechnology Information (https://www.ncbi.nlm.nih.gov/) GEO (Gene Expression Omnibus, https://www.ncbi.nlm.nih.gov/geo/) database is a completely international open resource containing large numbers of research chips and expression function sequencing data, as well as related experimental design data. We analysed the GSE118750 dataset using the hash function in Perl and the "limma" 20 R package to obtain the DEcircRNAs between OSCC and normal tissues (log fold change was set to 1.2). We then used the National Cancer Institute's Genomic Data Commons Data Portal to screen and download the "squamous cell neoplasms" that occurred in "other and unspecified parts of tongue, floor of mouth, other and unspecified parts of mouth, tonsil, base of tongue, Gum, oropharynx, palate, lip;” the results were used as our research data for OSCC. The original dataset contained 311 tumour cancer samples and 19 normal samples. Similarly, we used the “limma” package to analyse and obtain the DEmiRNAs and DEmRNAs (log fold change was set to 1.2). We drew heatmaps and volcano plots to visualize the differential expression of circRNAs, miRNAs, and mRNAs.

Using the Circ2Traits (http://gyanxet-beta.com/circdb/) database 21, we predicted the target miRNAs of the DEcircRNAs and obtained the intersection of these DEmiRNAs. We used FunRich (http://www.funrich.org/) 22 to predict the target mRNAs of the intersecting miRNAs and then obtained the intersection of these with the DEmRNAs in OSCC. We then constructed a ceRNA regulatory network map based on the target relationships among circRNAs, miRNAs, and mRNAs.

### 2.2 Biological pathway analysis

Using FunRich, we performed Kyoto Encyclopedia of Genes and Genomes 23 enrichment analysis on the 223 intersecting mRNAs. Using the STRING (https://string-db.org/) 24 database and Cytoscape 25, the interactions between the expression products of these mRNAs were plotted as a protein-protein interaction (PPI) network. Using the MCODE plug-in in Cytoscape (degree cut-off: 2; node score cut-off: 0.2; K-core: 2; max. depth: 100), two key submodules were found. We then used FunRich to obtain path enrichment results for these submodules.

### 2.3 Cox proportional hazard analysis and establishment of prognostic model

We selected 305 sequencing samples for which both mRNA and miRNA data were available from the 311 OSCC tumour samples obtained from TCGA; their information is shown in Table 1. Clinical samples with TNM stage information were included in the risk analysis. Samples with no specific clinical information were used for the analysis of bioinformatics data only. Using univariate Cox proportional hazard regression analysis, we screened and retained significant survival-related miRNAs and mRNAs (risk ratio: 95%; *P<*0.05). Using log-lambda in the Lasso algorithm as a penalty coefficient for regression analysis, we obtained the minimum value of the partial likelihood deviance. In the multivariate Cox proportional hazard regression analysis, the forest map of the risk assessment model was filtered according to the value of the Akaike information criterion. Finally, we identified the required mRNAs and miRNAs to establish a risk assessment prognosis model. We used receiver operating characteristic (ROC) curves to evaluate the selected biomarkers.

### 2.4 Analysis of immune infiltration

ESTIMATE is a tool for predicting the relationships between tumours and immune infiltration using an algorithm based on enrichment analysis of the sample gene set 26. We divided our 305 samples into two groups according to the risk score and performed ESTIMATE analysis to explore the relationship between the model and immune infiltration.

CIBERSORT (https://cibersort.stanford.edu/) 27 is an online tool for convoluting the expression matrix of human immune cell subtypes based on the principle of linear support vector regression. It provides a gene expression set of 22 human immune cell subtypes, which can be used to analyse cell abundance and gene expression in tissue samples. Using the R language to call the original dataset and the operation function of CIBERSORT, we obtained the proportions of 22 human immune cell subtypes in the target samples.

Tumour Immune Dysfunction and Exclusion (TIDE) was used to predict potential responses to immune checkpoint blockade (ICB) therapy based on the simulating tumour immune evasion mechanism 28. TIDE was used to predict the effects of ICB therapy in the high-risk and low-risk groups.

## 3. Results

### 3.1 Identification of differentially expressed circRNAs, miRNAs, and mRNAs

A total of 482 DEcircRNAs were identified from the GSE118750 dataset, comprising 475 high-expressed circRNAs and seven low-expressed circRNAs. From the OSCC TCGA data, we identified 92 DEmiRNAs (51 low-expressed miRNAs and 41 high-expressed miRNAs) and 961 DEmRNAs (421 low-expressed mRNAs and 540 high-expressed mRNAs). The corresponding heatmaps and volcano plots are shown in Fig. S1.

### 3.2 Construction of a ceRNA network in OSCC

**Fig 1:**
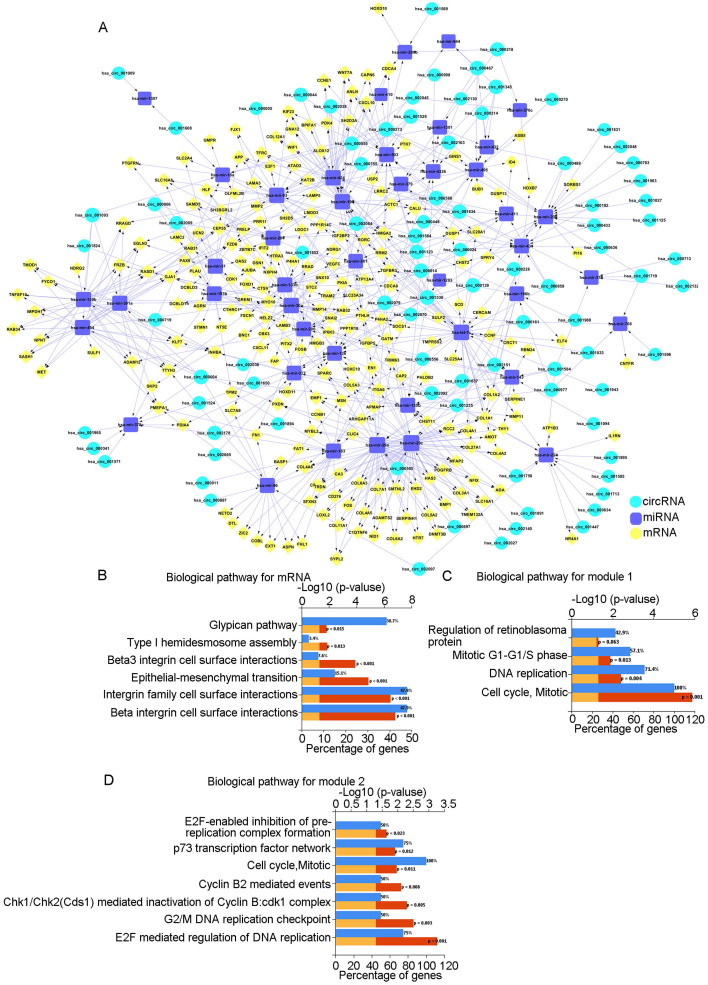
** ceRNA network and analysis of key modules.** A: The circRNA-miRNA-mRNA network in OSCC. Red represents circRNAs, green represents miRNAs, and yellow represents mRNAs. B: Biological pathways of mRNAs in the ceRNA network. C: Biological pathways of the first module. D: Biological pathways of the second module.

To further clarify the relationships among DEcircRNAs, DEmiRNAs, and DEmRNAs, we constructed a circRNA-miRNA-mRNA-related ceRNA network for OSCC. Using the Circ2Traits database, we identified 89 miRNAs that were targeted by DEcircRNAs and intersected these with the previously identified 92 DEmiRNAs to obtain 43 miRNAs. Then, we used FunRich to obtain mRNAs targeted by these 43 miRNAs and intersected these with the previously identified 961 DEmRNAs to obtain 223 mRNAs. We determined the associations between DEcircRNAs and DEmiRNAs and between DEmiRNAs and DEmRNAs (Supplement Table 1) and finally constructed a ceRNA network comprising 89 circRNAs, 43 miRNAs, and 223 mRNAs (Fig. 1A).

### 3.3 Biological pathway analysis

Biological pathway analysis of the 223 mRNAs showed that the pathways were mainly enriched in "beta1 integrin cell surface interactions," "integrin family cell surface interactions," and "epithelial-to-mesenchymal transition" (Fig. 1B). Using the STRING database and Cytoscape, we constructed a PPI network diagram. Using the MCODE plugin of Cytoscape, we selected two key modules. Pathway analysis showed that they were related to "Cell Cycle, Mitotic" and "E2F mediated regulation of DNA replication," among others (Fig. 1C, D).

### 3.4 Construction of the prognostic model

**Fig 2:**
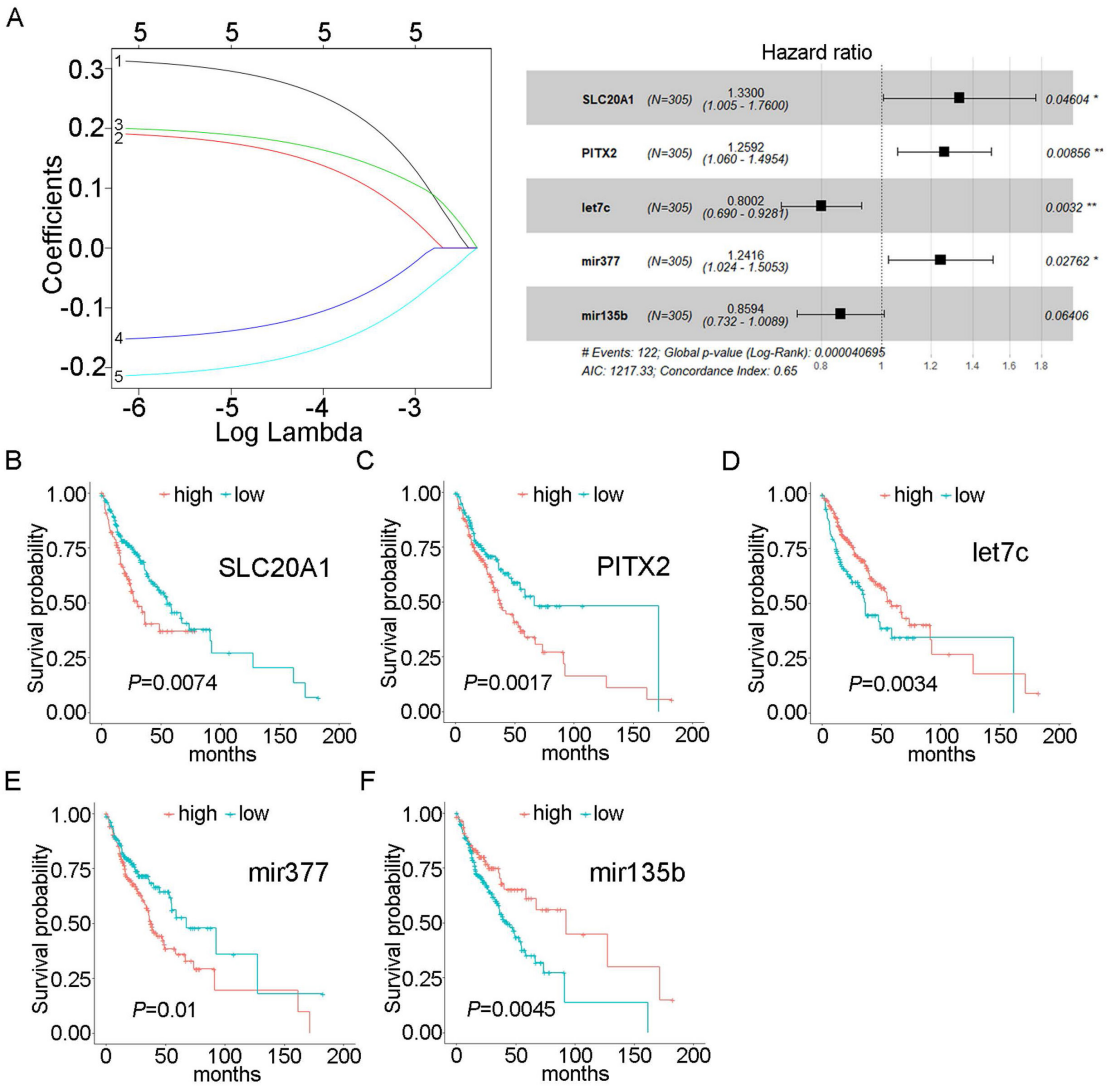
** Forest plot and survival analysis of biomarkers.** A: Cox proportional hazards model based on biomarkers. B: Survival analysis of SLC20A1 in OSCC (*P*=0.0074). C: Survival analysis of PITX2 in OSCC (*P*=0.0017). D: Survival analysis of hsa-let-7c in OSCC (*P=*0.0034). E: Survival analysis of hsa-miR-377 in OSCC (*P=*0.01). F: Survival analysis of hsa-miR-135b in OSCC (*P=* 0.0045).

Using univariate Cox proportional hazards regression analysis, we obtained 43 mRNAs and 6 miRNAs that were closely related to the survival of OSCC (*P*<0.05). After multivariate Cox proportional hazard regression analysis, two mRNAs and three miRNAs were retained and used to construct the prognostic model: SLC20A1, PITX2, hsa-mir-135b, hsa-mir-377, and hsa-let-7c (expression status is shown in Table 2). The risk score was calculated as follows: risk score = (0.28516×SLC20A1)+(0.23052×PITX2)+(-0.22286×hsa-let-7c)+(0.21641×hsa-mir-377)+(-0.15154×hsa-mir-135b). The concordance index for this model was 0.65 (Fig. 2A). The survival curves of the five biomarkers were consistent with the positive and negative coefficients in the formula (Fig. 2B-F). Survival curves were constructed using the Kaplan-Meier method and the log-rank test (Fig. S2A).

We validated the prognostic model using an external validation dataset from TCGA, selecting mRNA sequencing (mRNA-seq) and miRNA-seq data for OSCC (including laryngeal and hypopharyngeal cancers) and screening the matrix data for SLC20A1, PITX2, hsa-mir-135b, hsa-mir-377, and hsa-let-7c. A combined external dataset for OSCC containing 120 samples was obtained by analysing the information of clinical samples, and the risk score was calculated by substituting the score into the risk prognostic model (divided into high-risk and low-risk groups). Our results demonstrated that the prognostic model was suitable for use in OSCC (*P*<0.01) (Fig. S2B). The laryngeal cancer data were statistically significant (*P*<0.01) (Fig. S2C), while the hypopharyngeal cancer data were not statistically significant (*P*=0.11) (Fig. S2D).

### 3.5 Risk score and ROC curve analysis

**Fig 3:**
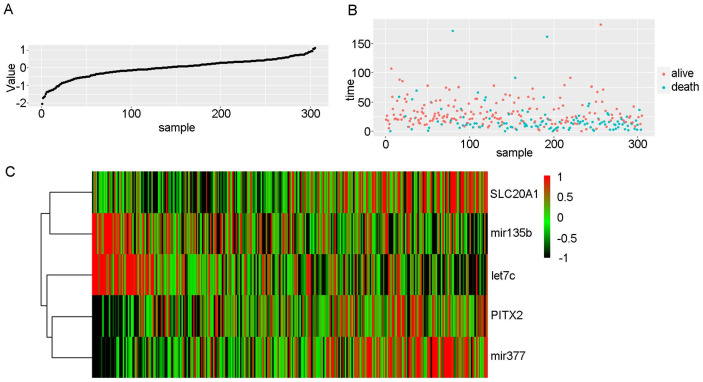
** Risk scores in OSCC.** A: Risk scores of OSCC patients in ascending order. B: Survival time and status of OSCC patients in order of increasing risk score. Red dots represent survival, and blue dots represent death. C: Heatmap showing the expression of these five biomarkers in OSCC in increasing order of risk score.

**Fig 4:**
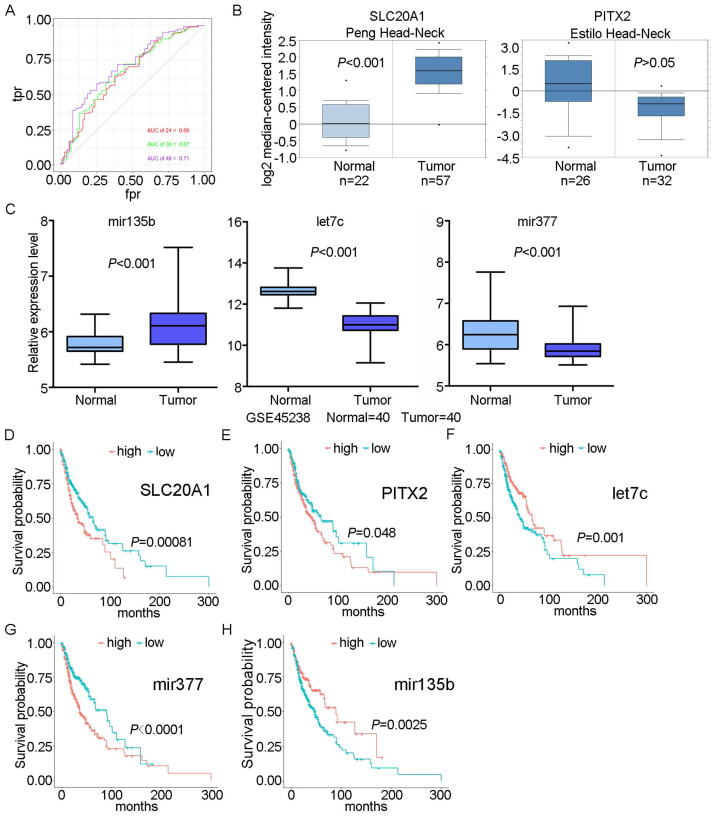
** ROC curve and survival analyses for biomarkers.** A: ROC curves for 2-, 3-, and 4-year survival with AUC values. B: Expression of SLC20A1 and PITX2 in the Oncomine database. C: Expression of hsa-let-7c, hsa-mir-377, and hsa-mir-135b in GSE45238. D: Survival analysis of SLC20A1 in head and neck squamous cell carcinoma (*P*= 0.00081). E: Survival analysis of PITX2 in head and neck squamous cell carcinoma (*P*= 0.048). F: Survival analysis of hsa-let-7c in head and neck squamous cell carcinoma (*P*= 0.001). G: Survival analysis of hsa-miR-377 in head and neck squamous cell carcinoma (*P*<0.0001). H: Survival analysis of hsa-miR-135b in head and neck squamous cell carcinoma (*P*= 0.0025).

We scored patients according to the prognostic model and arranged them in order from low to high risk. Fig. 3 shows the distribution of the patient risk score and the corresponding survival data, as well as the expression of the five biomarkers included in the prognostic model. Patients with high scores tended to have higher mortality. We also constructed a time-dependent ROC curve to evaluate these five biomarkers. The area under the ROC curve (AUC) values for the 2-, 3-, and 4-year survival rates were 0.66, 0.67, and 0.71, respectively (Fig. 4A). We also verified the expression levels of these five biomarkers in the Oncomine database (Fig. 4B) and in the GSE45238 dataset (Fig. 4C); except for PITX2 (for which no statistical significance was found, but the expression trend was consistent), the expression levels were consistent with those of the OSCC TCGA data (*P*<0.001). Then, we analysed the prognosis of these five biomarkers in head and neck squamous cell carcinoma data from TCGA and found that the prognosis was consistent with that found with the OSCC data (Fig. 4D-H). We also verified the random forest map, ROC curve, and risk assessment of this model in HNSC (head and neck squamous cell carcinoma) (Fig. S3).

To identify the differences in somatic mutations between the low-risk group and high-risk group, mutation data were visualized using the “maftools” R package 29. In the high-risk group, 79% (n = 105) of patients harboured a TP53 mutation, compared with 57% (n = 90) in the low-risk group (Fig. S4A). Moreover, PIK3CA, CDKN2A, FBXW7, and HRAS were found to be driver genes in the high-risk group; these genes may be closely related to the prognosis of patients (Fig. S4B, C).

### 3.6 Immune infiltration analysis in OSCC

Using the ESTIMATE algorithm, we evaluated the samples in the high-risk and low-risk groups and found that the low-risk samples had higher immune scores and lower stromal scores; the differences were statistically significant in both cases (Fig. S5), suggesting that the model may be related to immune infiltration.

**Fig 5:**
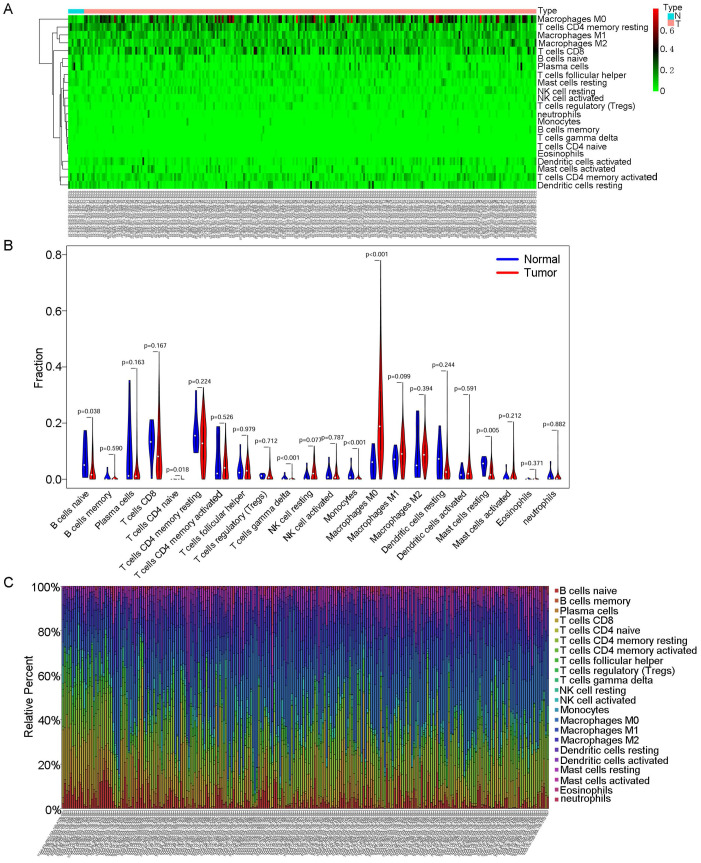
** CIBERSORT analyses in OSCC.** A: Heatmap of immune cell infiltration in OSCC samples and normal samples. Red represents OSCC samples, and blue represents normal tissue samples. B: Violin chart comparing the immune cells in OSCC samples and normal tissue samples. C: Bar chart with different colours representing different cell types arranged in ascending order according to the risk scores of the samples.

**Fig 6:**
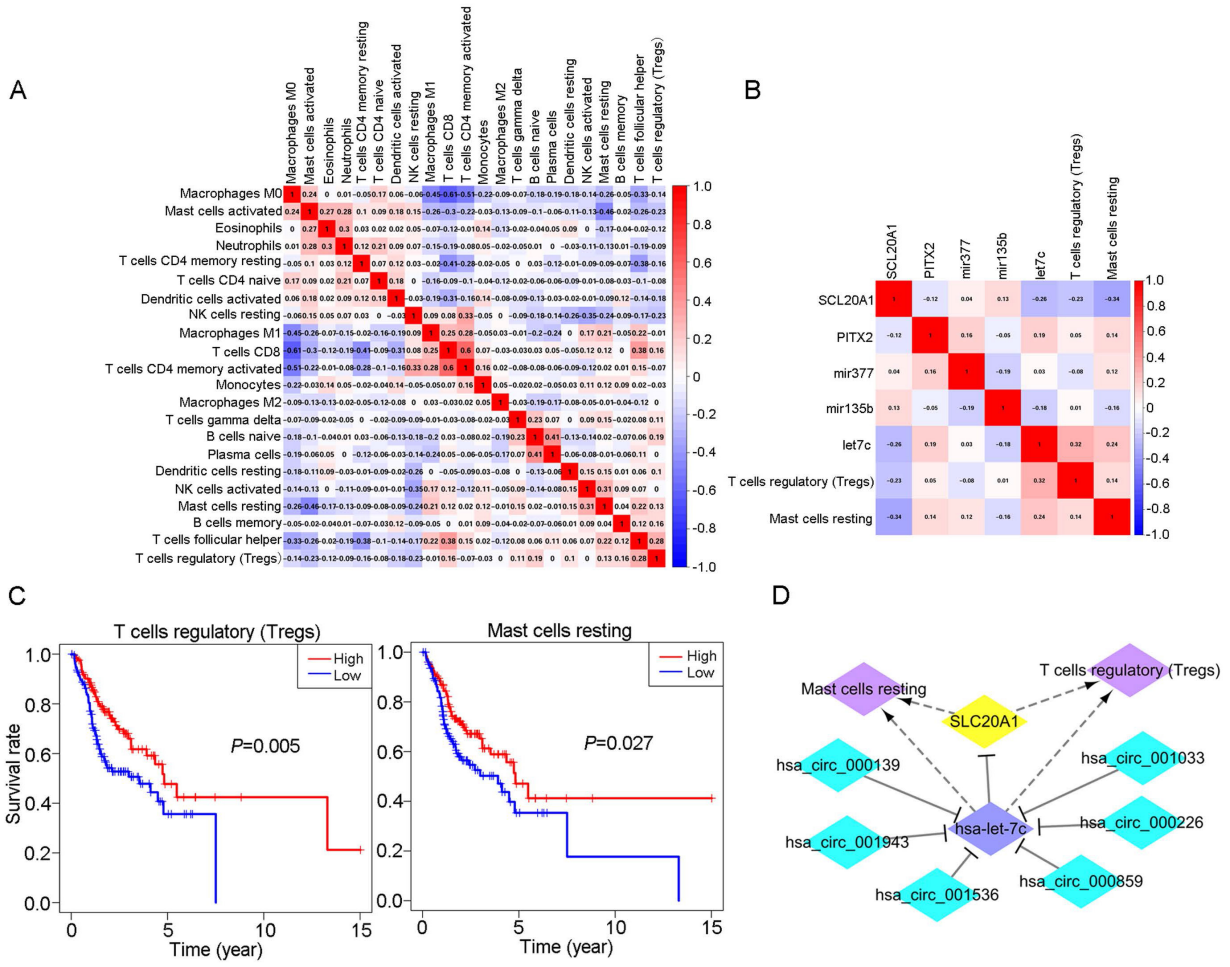
** Relationship between immune cells and biomarkers.** A: Correlation analysis of different immune cell types. B: Correlation analysis between different immune cell types and biomarkers. C: Survival analysis for Tregs and resting mast cells in OSCC (*P*<0.05). D: The ceRNA-immune cell network in OSCC.

Then, we used CIBERSORT to further explore the relationship between our model and immune infiltration in OSCC samples. As shown in the heatmap in Fig. 5A, more M0 macrophages were found in OSCC samples than in normal tissue samples. Tumour samples contained more macrophages and fewer B and T cells (Fig. 5B). We ranked the samples in order of risk from low to high and found that tumour samples classified as low risk tended to contain more B and T cells (Fig. 5C). In the survival analysis for the 22 immune cell types, we found that patients with higher levels of regulatory T cells (Tregs) and mast cells had a better prognosis (Fig. 6C).

TIDE was used to evaluate the therapeutic efficacy of immune checkpoint suppression in the low-risk and high-risk groups. The risk scores for CD8 and CTL (cytotoxic T lymphocytes) were higher in the low-risk group, indicating better prognosis (Fig. S6A, B). The scores for myeloid-derived suppressor cells (MDSCs) and cancer-associated fibroblasts (CAFs) were higher in the high-risk group, indicating less immune infiltration and poorer prognosis (Fig. S6C, D).

### 3.7 Comprehensive analysis of immune cells and biomarkers

We obtained coexpression relationships among various immune cells through correlation analysis (Fig. 6A) and then further analysed the correlations between immune cells and biomarkers (Fig. 6B). As shown in the figures, SLC20A1 expression levels were negatively correlated with the numbers of Tregs (R=-0.23, *P*<0.001) and resting mast cells (R=-0.34, *P*<0.001) (Fig. S7A), and hsa-let-7c expression was positively correlated with the numbers of Tregs (R=0.32, *P*<0.001) and resting mast cells (R=0.24, *P*<0.001) (Fig. S7B).

According to our ceRNA network, SLC20A1 was identified as a target gene of hsa-let-7c. SLC20A1 was highly expressed in tumour samples, whereas hsa-let-7c had low expression. Furthermore, in the ceRNA network, the six circRNAs (hsa_circ_000139, hsa_circ_001943, hsa_circ_001536, hsa_circ_000859, hsa_circ_000226, and hsa_circ_001033) targeting hsa-let-7c were all highly expressed in tumour samples, in good agreement with the endogenous competitive RNA hypothesis. Therefore, we constructed a ceRNA-immune cell network diagram (Fig. 6D). Using circBase (http://www.circbase.org/), we obtained proven target genes (YAF2, CLASP2, PDIA4, ETFA, POLR2A, and C1orf27) of these six circRNAs. Using GEPIA (http://gepia.cancer-pku.cn/index.html) website tools, we found that these six genes also had good positive correlations with SLC20A1 in head and neck squamous cell carcinoma (Fig. S8).

In summary, we established a circRNA-miRNA-mRNA network in OSCC through bioinformatics analysis. We identified SLC20A1, PITX2, hsa-mir-135b, hsa-mir-377, and hsa-let-7c as potential biomarkers for evaluating the prognosis of OSCC patients and established a prognostic model. Immune infiltration showed that the levels of M0, M1, and M2 macrophages were increased in OSCC samples, whereas the numbers of Tregs and resting mast cells were related to prognosis and had good correlations with specific biomarkers. It has been well established that the M1-to-M2 transition plays a part in tumour progression. Our results implied that increased numbers of M1 macrophages might be converted into M2 macrophages as reserve cells during OSCC progression 30. Thus, our research helps to elucidate the mechanism underlying OSCC and could indicate new strategies for immunotherapy.

## 4. Discussion

In this study, we first screened differentially expressed molecules between OSCC and normal samples by microarray analysis and constructed a ceRNA network comprising 89 circRNAs, 43 miRNAs, and 223 mRNAs to explore the mechanisms involving circRNAs in OSCC. Then, we analysed the biological pathways of the 223 mRNAs to explore the functions of circRNAs through their target mRNAs. Next, we identified two mRNAs (SLC20A1 and PITX2) and three miRNAs (hsa-mir-135b, hsa-mir-377, and hsa-let-7c) and used them to establish a prognostic model. Risk score and ROC curve analyses supported the reliability of the five biomarkers and the prognostic model. Finally, we carried out immune infiltration analysis and comprehensive analysis of immune cells and biomarkers in OSCC. We found that OSCC had a unique immune microenvironment, with good correlations between immune cells and biomarkers. Our study thus elucidates the pathogenesis of OSCC based on the ceRNA network, proposes potential biomarkers and prognostic models, and provides new ideas for immunotherapy for OSCC patients.

Our functional enrichment analysis showed that the 233 genes were mainly related to the "Beta1 integrin cell surface interactions," "Integrin family cell surface interactions," "Epithelial-to-mesenchymal transition," and "Beta3 integrin cell surface interactions Beta3" pathways. We clustered our large PPI network graph to obtain functional modules and found that there were two key modules: "Cell Cycle, Mitotic cell cycle, mitosis" and "E2F mediated regulation of DNA replication." Integrin is a transmembrane receptor on the cell surface that mediates cell adhesion to other cells or the extracellular matrix and participates in multiple signalling pathways. Specific changes in integrin are common in tumours, promoting tumour migration and survival in different environments 31. A specific integrin, αvβ6, has been found to promote the development of OSCC 32. Many studies have emphasized the importance of epithelial-mesenchymal transformation (EMT) in tumour invasion and metastasis. Our results suggest that integrin and EMT may be key factors in the high invasiveness and metastasis of OSCC and that circRNAs may participate in the occurrence and development of OSCC through multiple pathways.

Hsa-mir-135b has been widely studied in many tumour types and is highly expressed in colon and gastric cancer tissues. The Wnt and PI3K/AKT signalling pathways promote the transcriptional activation of hsa-mir-135b, whereas hsa-mir-135b can in turn affect the activity of the Wnt pathway to promote the proliferation and invasion of tumour cells 33, 34. Hsa-mir-135b also promotes the growth of blood vessels in tumour tissue by inhibiting the expression of FOXO1 protein 35. Previous bioinformatics studies have found that hsa-mir-135b is highly expressed in OSCC 36, 37, and our study further confirmed this conclusion. However, we also found that higher expression of hsa-mir-135b in tumour tissue was associated with better prognosis. Further verification using UCSC Xena (https://xenabrowser.net/) showed that pancancer patients with high expression of hsa-miR-135b had a better prognosis (Fig. S9A) (*P<*0.05). This finding indicates a protective effect of hsa-mir-135b. Our results thus provide a new perspective on the role of hsa-miR-135b in tumours, which needs to be further explored. Hsa-miR-377 has an inhibitory role in the development and invasion of multiple cancers. Hsa-mir-377 targets ETS1 to inhibit human clear cell renal cell carcinoma 38, targets E2F3 to inhibit non-small cell lung cancer 39, and targets BRD4 to inhibit colon cancer 40. Our results showed low expression of hsa-mir-377 in OSCC. Interestingly, we found that higher expression of hsa-miR-377 corresponded to a worse prognosis; this was verified on a pancancer basis (*P<*0.01) (Fig. S9B). Hsa-miR-377 thus seemed to be conducive to tumour development, indicating a new perspective on its role in tumours.

Hsa-let-7c acts as a tumour suppressor by targeting N-RAS, C-MYC, MMP1, PBX2, PBX3, and other factors 41. Hsa-let-7c also targets IGF-1R through the MAPK pathway to inhibit the directional differentiation of dental pulp mesenchymal stem cells 42. Our study found low expression of hsa-let-7c in OSCC; the higher the expression of hsa-let-7c in tumour tissue, the better the prognosis. SLC20A1 encodes sodium-dependent phosphate transporter 1 (PiT1). PiT1 is responsible for the activation of NF-κB, an important tumour-promoting pathway, and PiT-1 is directly related to cell proliferation 43. SLC20A1 activates the Wnt/β-catenin signalling pathway and promotes the growth, invasion, and recurrence of growth hormone adenomas 44. We found that SLC20A1 was highly expressed in OSCC and associated with worse prognosis. The coding product of the paired-like homeodomain transcription Factor 2 (PITX2) gene acts as a transcription factor. In addition to being physiologically involved in the development of the anterior structure, PITX2 promotes the development of multiple tumours and improves their drug resistance 45. Our study showed low expression of PITX2 in OSCC, with its content in tumour tissue negatively correlated with prognosis.

The immune infiltration analysis showed significant increases in the numbers of M0, M1, and M2 macrophages in OSCC compared with normal tissues. Macrophages are important components of the tumour microenvironment. M1-like macrophages appear in the initial stage of tumour development and secrete proinflammatory cytokines, whereas M2-like macrophages are predominant in the metastasis stage and secrete anti-inflammatory factors. The M1-to-M2 transition plays a part in tumour progression 25; thus, increased numbers of M1 macrophages may transform to M2 macrophages to promote OSCC progression. Therefore, the polarization of macrophages has a vital role in tumours 46.

In recent years, the relationship between PD-1/PD-L1 and macrophages has attracted increasing attention. PD-1 is an inhibitory receptor expressed on B cells, dendritic cells, macrophages, and T cells, and its expression on macrophages increases with the progression of disease. PD-L1 is a ligand of PD-1 that is widely expressed in many cell types. It is well known that PD-1 and PD-L1 mediate immune suppression in tumours by inhibiting T-cell activation. Recent studies have shown that PD-1 also inhibits phagocytosis in macrophages and may induce M2 polarization. Macrophage polarization is a potential tumour inhibition mechanism induced by anti-PD-1 therapy, and macrophages may also be responsible for nonresponse or drug resistance in patients treated with anti-PD-1 therapy 47. Our results provide a further theoretical basis for the mechanism of anti-PD-1 targeted therapy in OSCC. A combination of PD-1/PD-L1 blockade and macrophage-targeting therapy may have a better antitumour effect. Our results also show that the higher the proportions of Tregs and resting mast cells in tumour tissues are, the better the prognosis of the patient.

Mast cells are secretory cells residing in tissues; they have important roles in innate and adaptive immunity and participate in chronic inflammation and tumour development. Activated mast cells secrete inflammatory cytokines, mainly to promote tumour growth, whereas a higher proportion of resting mast cells may correspond to a reduced inflammatory response, indicating a better prognosis 48. It is generally believed that Treg cells promote tumour development by inhibiting antitumour immunity. However, in cancers characterized by significant chronic inflammation, such as colon, breast, bladder, or head and neck cancer 49, higher Treg infiltration levels within the tumour seem to correspond to better prognosis. This could be explained by a gene expression signature 50 or its capability to inhibit tumour-promoting inflammation 51. Our results enrich the current view of Tregs and provide evidence for Tregs as "good citizens" in tumours. The correlation analysis between immune cells and biomarkers showed that SLC20A1 and hsa-let-7c had good correlations with Tregs and resting mast cells, respectively. Our results suggest the regulatory potential of a circRNA network in immune cells.

Our research had some limitations. First, the size of the sample we obtained from public databases was limited, which may have caused random errors. Second, the heterogeneity of the immune microenvironment at different sites of tumour invasion was not taken into consideration. In addition, further experiments are needed to verify the interactions among circRNAs, miRNAs, and mRNAs and to further clarify their mechanisms.

In summary, we established a circRNA-miRNA-mRNA network in OSCC through bioinformatics analysis. We identified SLC20A1, PITX2, hsa-mir-135b, hsa-mir-377, and hsa-let-7c as potential biomarkers for evaluating the prognosis of OSCC patients and established a prognostic model. Immune infiltration analysis showed that M0, M1, and M2 macrophage levels were increased in OSCC, whereas those of Tregs and resting mast cells were related to prognosis and had good correlations with specific biomarkers. Our results help to elucidate the mechanisms underlying OSCC and indicate potential new strategies for immunotherapy.

## Supplementary Material

Supplementary figures and tables.

## Figures and Tables

**Table 1:** Baseline characteristics of 305 OSCC samples

Characteristic	Number of cases (%)
Age (years)	
≥60	149 (48.9)
<60	156 (51.1)
Total	305
Gender	
Male	220 (72.1)
Female	85 (27.9)
Total	305
Clinical stage	
Ⅰ	15 (5.1)
Ⅱ	62 (21.0)
Ⅲ	57 (19.3)
Ⅳ	161 (54.6)
Total	305
T classification	
T1	28 (9.2)
T2	97 (31.8)
T3	79 (25.9)
T4	90 (29.5)
Unknown	11 (3.6)
Total	305
N classification	
N0	136 (44.6)
N1	48 (15.7)
N2	104 (34.1)
N3	3 (1.0)
Unknown	14 (4.6)
Total	305
Metastasis	
No	283 (92.8)
Yes	3 (1.0)
Unknown	19 (6.2)
Total	305

**Table 2:** Expression status of biomarkers

Biomarker	logFC	*P value*	Change direction
SLC20A1	1.236585	7.36E-16	Up
PITX2	-1.26913	9.53E-08	Down
hsa-mir-135b	1.766621	7.49E-09	Up
hsa-mir-377	-1.51776	3.16E-10	Down
hsa-let-7c	-2.16479	1.85E-10	Down

## Abbreviations

Abbreviations and Full Names of all concepts in this manuscript can be found in the Supplementary Material (Supplement Table 2).
