# Supplementary material for: Identification of a CircRNA-miRNA-mRNA Network and Integrated Analysis of Immune Infiltration in Oral Squamous Cell Carcinoma
Source: J Cancer. 2023 Jan 1;14(2):250–61. doi: 10.7150/jca.79967 (PMC9891867; doi:10.7150/jca.79967)
Supplement: Supplementary file 1 — Supplementary figures and tables. [file jcav14p0250s1.pdf]

Supplementary Figures

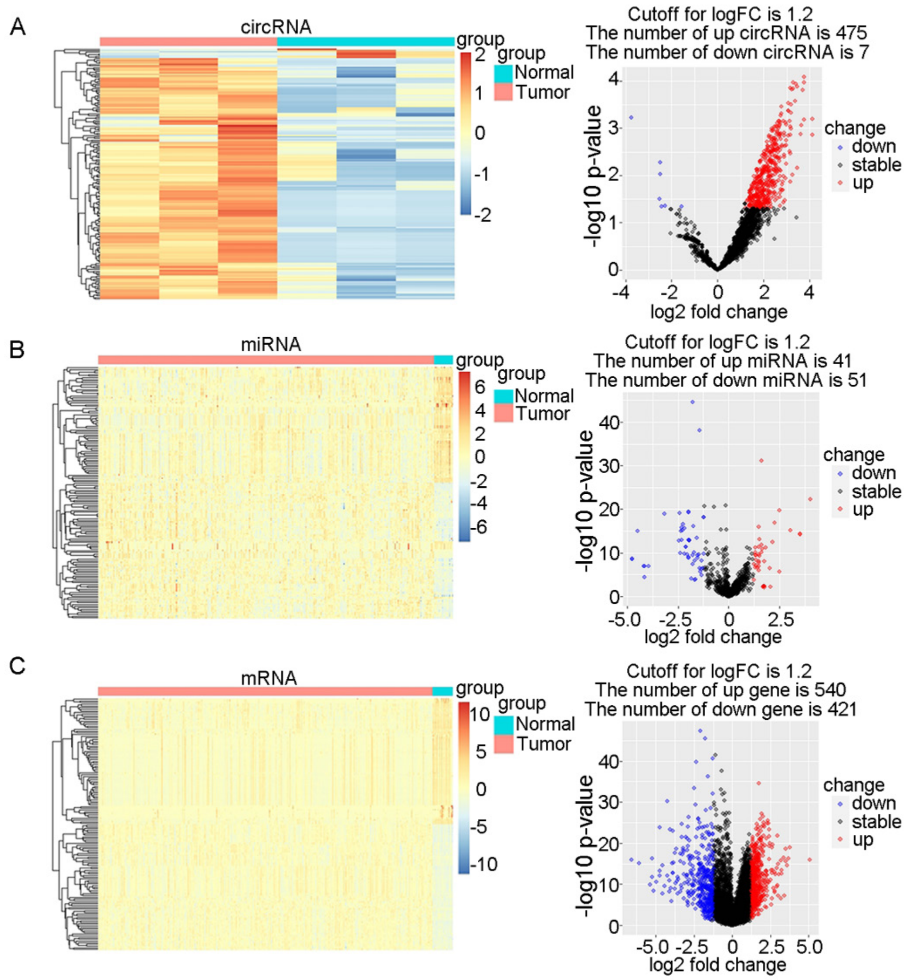

Fig. S1 Identification of biomarkers

In the heatmap, blue indicates low expression, and red indicates high expression; in the volcano plot, blue dots represent the expression of biomarkers with *P*<0.05 and log2 fold change (FC) <1.2. Red plots represent the expression of biomarkers with *P*< 0.05 and log2 FC>1.2. Black plots represent genes that expressed biomarkers normally. The X-axis indicates the log2 FC of the expression of biomarkers between normal and tumour samples. The Y-axis indicates the log10 *P value* for each biomarker. A: Heatmap and volcano plot of circRNAs between OSCC and normal samples from GSE118750. B: Heatmap and volcano plot of miRNAs between OSCC and normal samples from TCGA. C: Heatmap and volcano plot of mRNAs between OSCC and normal samples from TCGA.

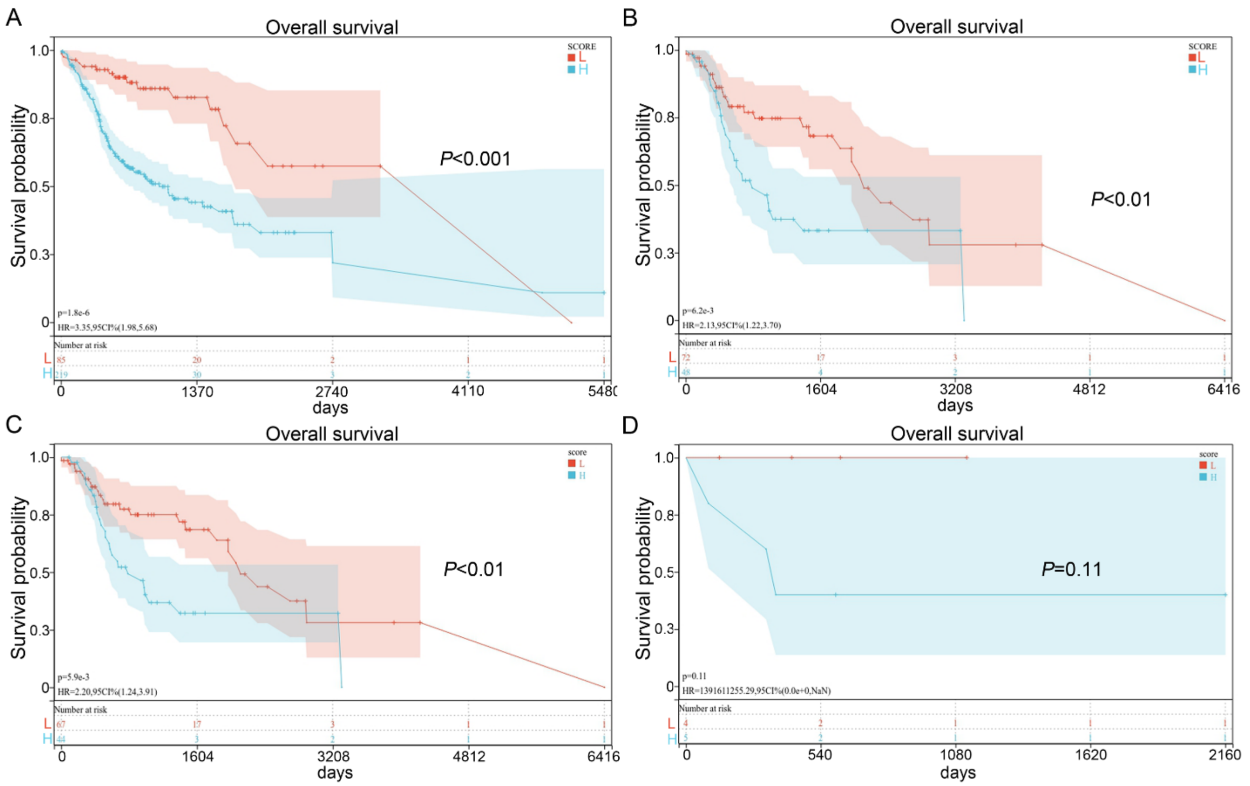

Fig. S2 Survival analysis of the external validation dataset from TCGA

The matrix and clinical information from the original oral cancer cohort were substituted into the risk score formula, and the obtained risk scores were grouped by the optimal cut-off value given by the R package maxstat; L represents the low-risk group, and H represents the high-risk group. A. The results of the multigroup comparison analysis based on the log-rank test; P=1.8e-6<0.01, HR=3.35, 95 CI% (1.98, 5.68). B. Data for the 120 external cohorts containing matrix and clinical information from TCGA were input into the formula of the prognostic model; the results of the multigroup comparison analysis based on the log-rank test are shown; P=6.2e-3<0.01, HR=2.13, 95 CI% (1.22, 3.70). C. The results of the survival analysis of the laryngeal cancer data were statistically significant, P=5.9e-3<0.01, HR=2.20, 95% CI (1.24, 3.91). D. The results of the survival analysis of hypopharyngeal cancer were not statistically significant (P=0.11).

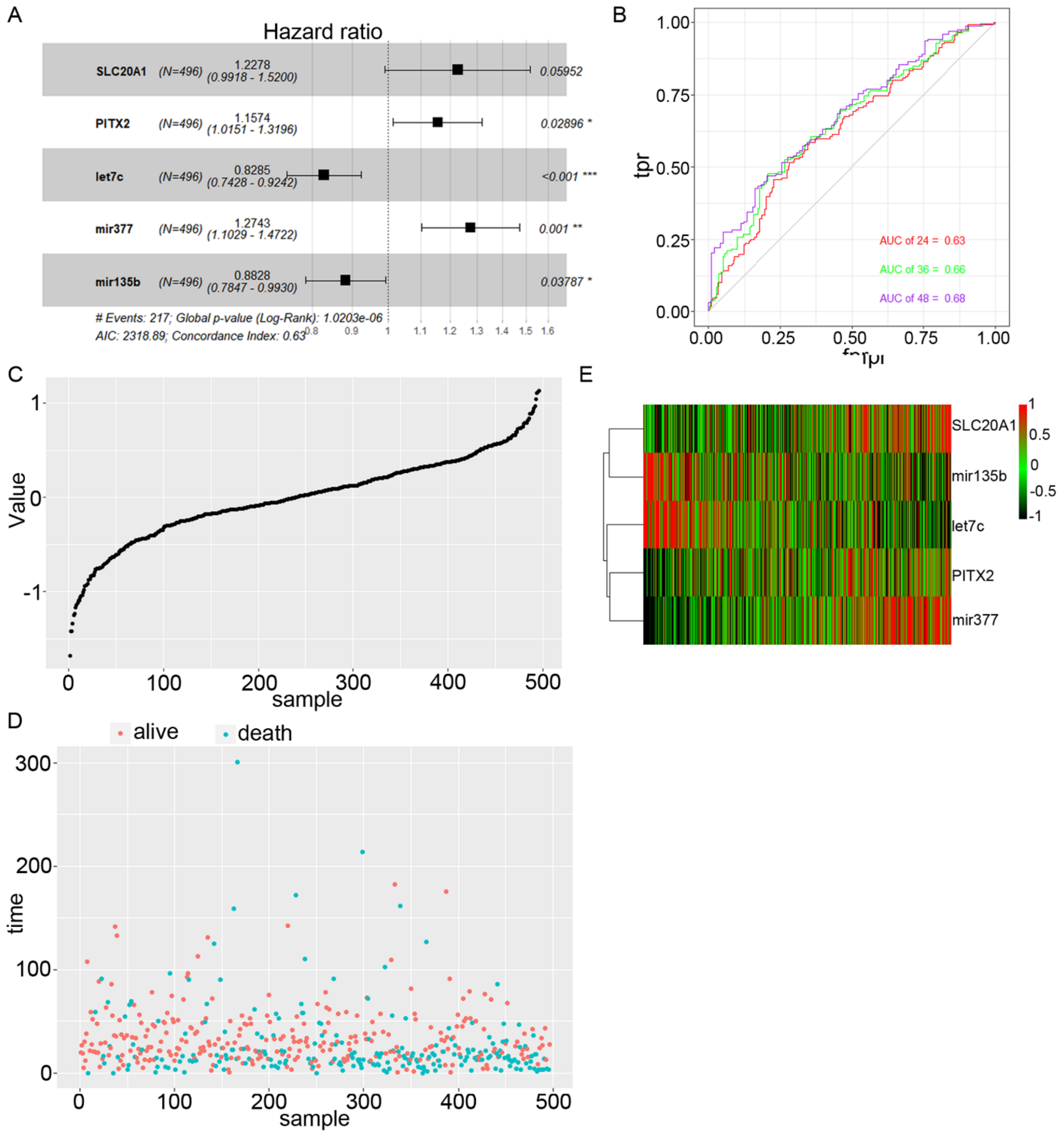

Fig. S3 Relationship between five biomarkers and risk scores of HNSC patients.

A: Forest plot of five biomarkers in HNSC. B: ROC curves for 2-, 3-, and 4-year survival with AUC values in HNSC. C: Risk scores of HNSC patients in ascending order. D: Survival times and status of HNSC patients in order of increasing risk score. Red dots represent survival, and blue dots represent death. E: Heatmap showing the expression of these five biomarkers in HNSC in order of increasing risk score.

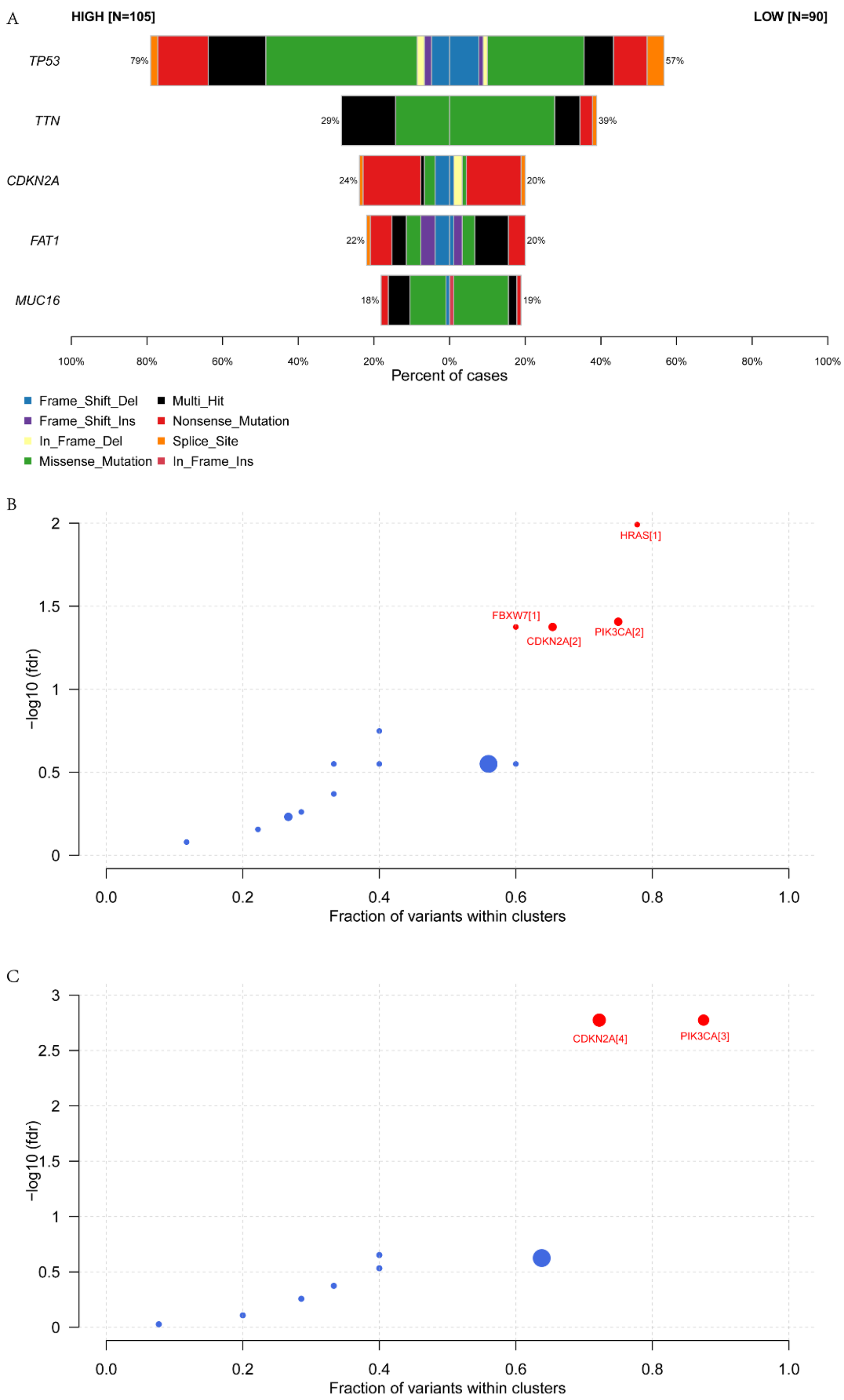

Fig. S4 Somatic mutation analyses

A: Differences in mutant genes between the high-risk group and the low-risk group. B: Cancer driver genes in the high-risk group. C: Cancer driver genes in the low-risk group.

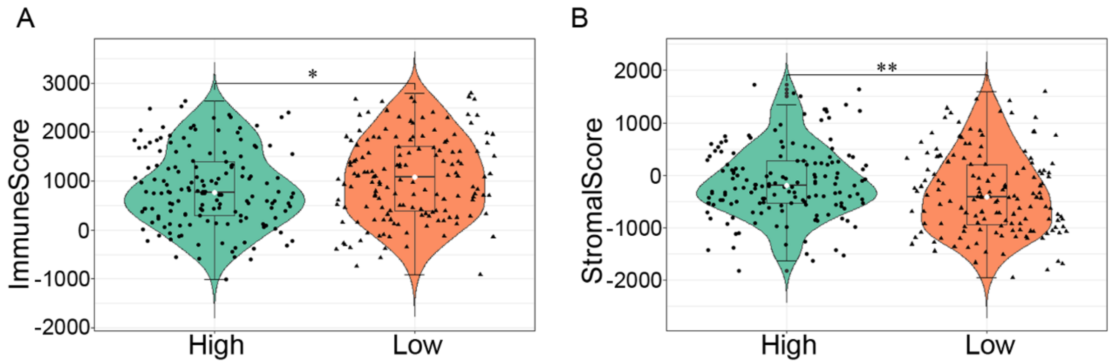

Fig. S5 Immune scores and stromal scores for high-risk and low-risk samples.

A: Immune scores of high-risk and low-risk samples obtained by ESTIMATE. B: Stromal scores for high-risk and low-risk samples obtained by ESTIMATE.

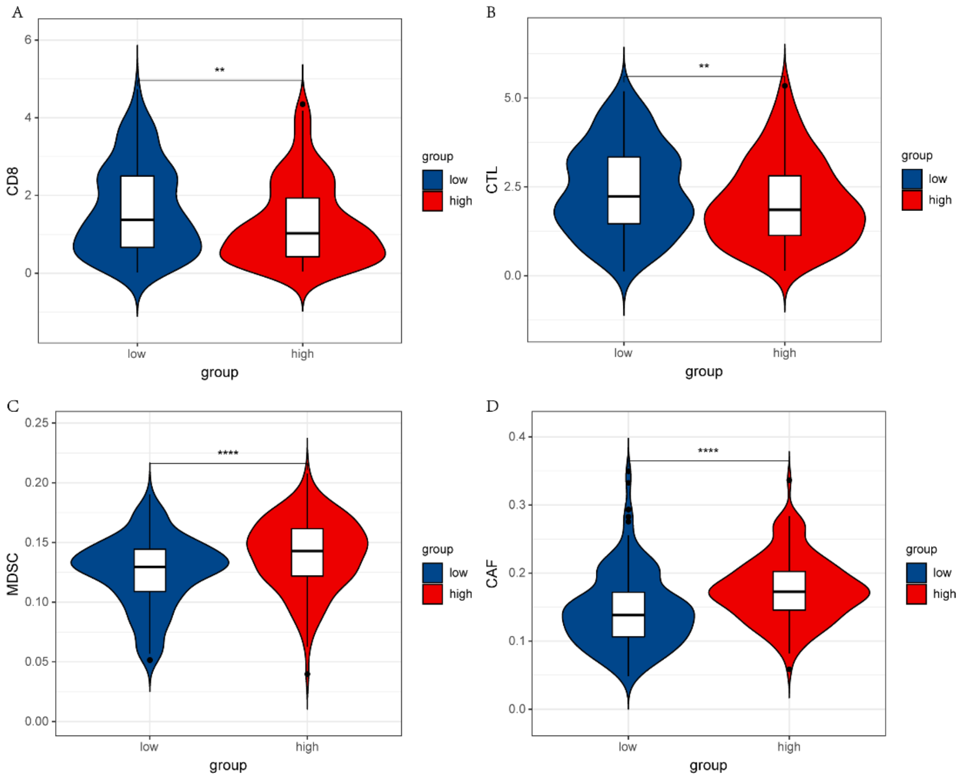

Fig. S6 TIDE analysis

A: CD8 scores for high-risk and low-risk samples. B: CTL scores for high-risk and low-risk samples. C: MDSC scores for high-risk and low-risk samples. D: CAF scores for high-risk and low-risk samples.

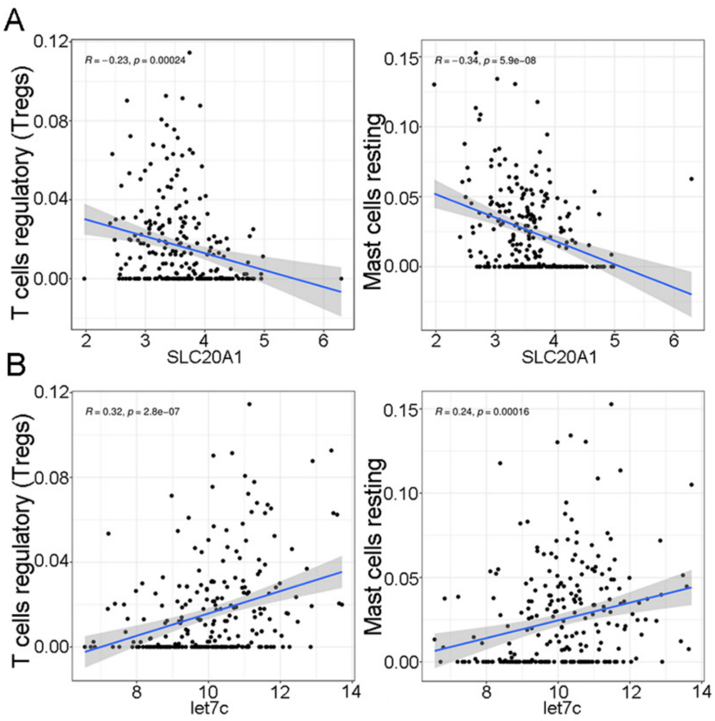

Fig. S7 Correlation analysis for biomarkers and immune cells

A: Analysis of the correlations of SLC20A1 with Tregs and resting mast cells B: Analysis of the correlations of hsa-let-7c with Tregs and resting mast cells.

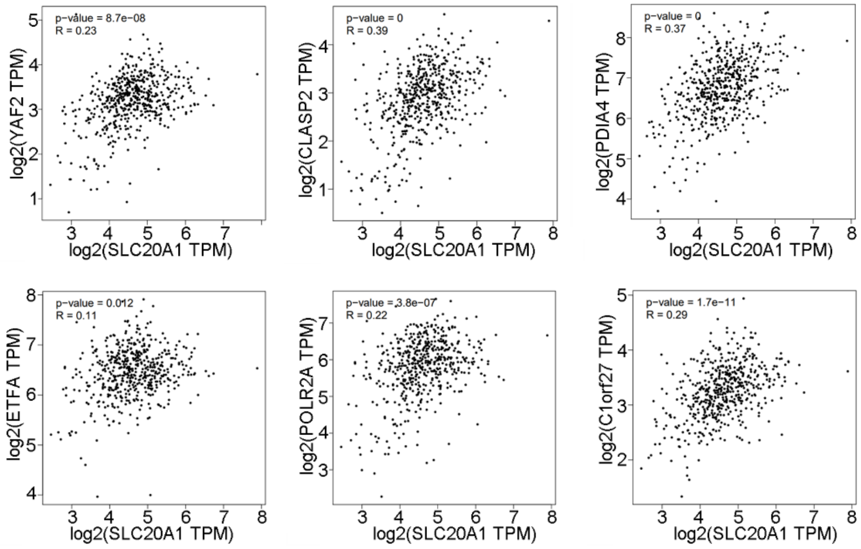

Fig. S8 Correlation analysis for SLC20A1 and target genes of six circRNAs in head and neck squamous cell carcinoma.

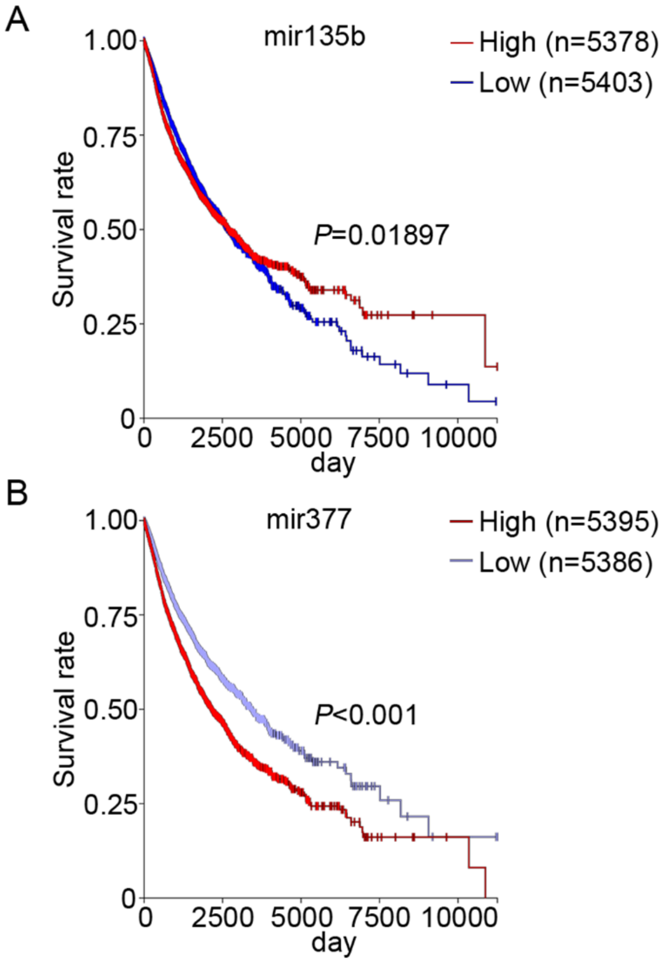

Fig. S9 Pancancer survival analysis for miRNAs

A: Pancancer survival analysis of hsa-miR-135b (*P*<0.05). B: Pancancer survival analysis of hsa-miR-377 (*P*<0.01).

Supplementary Tables

Supplementary Table 1

Associations between DEcircRNAs and DEmiRNAs

circRNA	miRNAs
hsa_circ_000004	hsa-mir-96; hsa-mir-454; hsa-mir-378c; hsa-mir-301a; hsa-mir-135b; hsa-mir-130b
hsa_circ_000005	hsa-mir-93
hsa_circ_000006	hsa-mir-31; hsa-mir-204; hsa-mir-193b
hsa_circ_000011	hsa-mir-183
hsa_circ_000014	hsa-mir-503; hsa-mir-424; hsa-mir-206; hsa-mir-205; hsa-mir-195; hsa-mir-143; hsa-mir-135b
hsa_circ_000024	hsa-mir-431; hsa-mir-133b
hsa_circ_000044	hsa-mir-503; hsa-mir-424; hsa-mir-195
hsa_circ_000139	hsa-mir-206; hsa-mir-205; hsa-mir-136; hsa-let-7c
hsa_circ_000161	hsa-mir-708; hsa-mir-30a; hsa-mir-136
hsa_circ_000182	hsa-mir-431
hsa_circ_000218	hsa-mir-376c; hsa-mir-208b; hsa-mir-1301
hsa_circ_000226	hsa-mir-379; hsa-mir-196b; hsa-let-7c
hsa_circ_000270	hsa-mir-495
hsa_circ_000273	hsa-mir-944; hsa-mir-93; hsa-mir-495; hsa-mir-4326; hsa-mir-410; hsa-mir-381; hsa-mir-208b; hsa-mir-205; hsa-mir-18a
hsa_circ_000314	hsa-mir-4326; hsa-mir-432
hsa_circ_000341	hsa-mir-378c
hsa_circ_000433	hsa-mir-431; hsa-mir-205
hsa_circ_000448	hsa-mir-431; hsa-mir-204
hsa_circ_000467	hsa-mir-944; hsa-mir-495; hsa-mir-432; hsa-mir-379
hsa_circ_000489	hsa-mir-432; hsa-mir-431; hsa-mir-205
hsa_circ_000505	hsa-mir-29c; hsa-mir-29a
hsa_circ_000555	hsa-mir-204; hsa-mir-18a; hsa-mir-1301
hsa_circ_000556	hsa-mir-206; hsa-mir-143
hsa_circ_000568	hsa-mir-503; hsa-mir-431; hsa-mir-424; hsa-mir-411; hsa-mir-195;
hsa_circ_000634	hsa-mir-224
hsa_circ_000636	hsa-mir-431
hsa_circ_000713	hsa-mir-375
hsa_circ_000719	hsa-mir-378c; hsa-mir-18a
hsa_circ_000755	hsa-mir-4326; hsa-mir-133b
hsa_circ_000783	hsa-mir-205
hsa_circ_000859	hsa-mir-431; hsa-mir-411; hsa-let-7c
hsa_circ_000887	hsa-mir-96
hsa_circ_000897	hsa-mir-29c; hsa-mir-29a
hsa_circ_000977	hsa-mir-708; hsa-mir-196b; hsa-mir-135b
hsa_circ_000998	hsa-mir-4326
hsa_circ_001027	hsa-mir-205
hsa_circ_001033	hsa-let-7c
hsa_circ_001093	hsa-mir-454; hsa-mir-301a; hsa-mir-18a; hsa-mir-130b
hsa_circ_001094	hsa-mir-143
hsa_circ_001123	hsa-mir-432; hsa-mir-411; hsa-mir-183; hsa-mir-1301
hsa_circ_001125	hsa-mir-205
hsa_circ_001235	hsa-mir-224; hsa-mir-136
hsa_circ_001345	hsa-mir-944; hsa-mir-495; hsa-mir-432; hsa-mir-379
hsa_circ_001447	hsa-mir-224

hsa_circ_001524	hsa-mir-378c; hsa-mir-136
hsa_circ_001529	hsa-mir-503; hsa-mir-432; hsa-mir-424; hsa-mir-195
hsa_circ_001536	hsa-mir-503; hsa-mir-4326; hsa-mir-424; hsa-mir-377; hsa-mir-224; hsa-mir-206; hsa-mir-205; hsa-mir-195; hsa-mir-143; hsa-mir-136; hsa-mir-135b; hsa-mir-1293; hsa-let-7c
hsa_circ_001564	hsa-mir-93; hsa-mir-495; hsa-mir-431; hsa-mir-381; hsa-mir-136
hsa_circ_001571	hsa-mir-378c
hsa_circ_001584	hsa-mir-375; hsa-mir-224; hsa-mir-143; hsa-mir-135b
hsa_circ_001585	hsa-mir-224
hsa_circ_001589	hsa-mir-208b
hsa_circ_001596	hsa-mir-708
hsa_circ_001608	hsa-mir-18a; hsa-mir-1307
hsa_circ_001637	hsa-mir-29c; hsa-mir-29a; hsa-mir-205
hsa_circ_001650	hsa-mir-136
hsa_circ_001713	hsa-mir-224
hsa_circ_001719	hsa-mir-708; hsa-mir-375; hsa-mir-205
hsa_circ_001758	hsa-mir-224; hsa-mir-135b
hsa_circ_001824	hsa-mir-193b
hsa_circ_001831	hsa-mir-205
hsa_circ_001834	hsa-mir-503; hsa-mir-424; hsa-mir-375; hsa-mir-195
hsa_circ_001852	hsa-mir-381; hsa-mir-206
hsa_circ_001891	hsa-mir-135b
hsa_circ_001894	hsa-mir-136
hsa_circ_001895	hsa-mir-224
hsa_circ_001903	hsa-mir-205
hsa_circ_001909	hsa-mir-1307
hsa_circ_001943	hsa-let-7c
hsa_circ_001965	hsa-mir-378c
hsa_circ_001988	hsa-mir-196b
hsa_circ_002009	hsa-mir-30a; hsa-mir-204
hsa_circ_002027	hsa-mir-135b
hsa_circ_002036	hsa-mir-136
hsa_circ_002038	hsa-mir-503; hsa-mir-424; hsa-mir-195
hsa_circ_002045	hsa-mir-93; hsa-mir-376c
hsa_circ_002048	hsa-mir-205
hsa_circ_002064	hsa-mir-503; hsa-mir-4326; hsa-mir-424; hsa-mir-195; hsa-mir-136
hsa_circ_002065	hsa-mir-377
hsa_circ_002070	hsa-mir-495; hsa-mir-183
hsa_circ_002079	hsa-mir-503; hsa-mir-4326; hsa-mir-424; hsa-mir-411; hsa-mir-377; hsa-mir-29c; hsa-mir-29a; hsa-mir-195; hsa-mir-136
hsa_circ_002092	hsa-mir-377; hsa-mir-30a; hsa-mir-224
hsa_circ_002097	hsa-mir-96; hsa-mir-224
hsa_circ_002103	hsa-mir-93; hsa-mir-205; hsa-mir-1301
hsa_circ_002130	hsa-mir-4326
hsa_circ_002132	hsa-mir-375
hsa_circ_002140	hsa-mir-135b
hsa_circ_002151	hsa-mir-431; hsa-mir-29c; hsa-mir-29a
hsa_circ_002178	hsa-mir-378c; hsa-mir-183; hsa-mir-136

Associations between DEmiRNAs and DEmRNAs

miRNA	mRNA
hsa-mir-495	BUB1; ASB5; ACTC1
hsa-mir-431	CHST2

hsa-mir-708	CNTFR
hsa-mir-376c	HOXB7
hsa-mir-208b	HOXD10; CDCA4
hsa-mir-224	NR4A1; IL1RN; ATP1B3
hsa-mir-135b	PHLDB2; MMP11; INHBA; EMP1; CHST11; CAP2; ARHGAP11A; APMAP;
hsa-mir-377	PKIA; PITX2; PDIA4; KLF7; ITGA6; HOXD11
hsa-mir-136	PPP1R18; HOXC10
hsa-mir-18a	PTGFRN; OLFML2B; IGF2BP2; GMPR
hsa-mir-378c	SKP2; PDIA4
hsa-mir-379	SLC20A1; MYO10; LRRC2; ID4; DUSP1
hsa-mir-93	SLC2A4; SLC16A6; SH2D5; RRM2; RRAGD; RORC; RASD1; PTHLH; PKIA; MMP2; LAMP5; LAMA3; KIF23; KAT2B; HMGA2; HLF; GJA1; GINS1; FJX1; EGLN3; E2F1; ATAD2; APP
hsa-mir-30a	SOCS1; SNX10; SLC25A34; SKP2; RRAD; RASD1; RAB32; PPP1R14C; P4HA2; P4HA1; MYBL2; ITGA6; IP6K3; HTRA3; HMGB3; HLF; GJA1; GATM; FRZB; FOXD1; FAP; DCBLD1; CTHRC1; CHST2; CBX3; CALU; BNC1; ACTC1
hsa-mir-205	SORBS1; PI16; GINS1; CALU
hsa-mir-411	SPRY4; ID4; DUSP13; DUSP1
hsa-mir-206	STC2; SNAI2; HELZ2; GJA1; FOSB; FN1; CXCL11; ADAM12
hsa-mir-196b	TGFBR3; RCC2; HOXB7; HMGA2; ELF4; COL1A2; COL1A1
hsa-mir-143	THY1; SERPINE1; RBM24; ITGA6; IGFBP5; COL1A1
hsa-let-7c	TMPRSS2; TGFBR3; SULF2; SOCS1; SLC25A4; SLC20A1; SCD; RRM2; RORC; PKIA; P4HA2; MSN; MMP11; IGF2BP2; HMGA2; GATM; ELF4; DUSP1; CRCT1; COL4A6; COL4A2; COL4A1; COL27A1; COL1A2; COL1A1; CERCAM; CDCA8; CCNF; AMOT
hsa-mir-193b	TPM2; TGFBR3; STMN1; SLC16A6; PLAU; NT5E; LAMC2; KLF7; IGFBP5; GREM1; AJUBA
hsa-mir-381	TRIM63; PPP1R14C; EN1
hsa-mir-29a	TRIM63; TMEM132A; SYPL2; SPARC; SMTNL2; SLC16A1; SFXN3; SERPINH1; RCC2; PXDN; PTHLH; PDGFRB; NID1; NFIX; MYBL2; MFAP2; LOXL2; HTR7; HAS3; FOS; EN1; EMP1; EHD2; DNMT3B; CSPG4; COL7A1; COL6A3; COL6A2; COL5A3; COL5A2; COL4A6; COL4A5; COL4A2; COL4A1; COL3A1; COL27A1; COL1A2; COL1A1; COL11A1; CD276; CA3; C1QTNF6; BMP1; AMOT; ADAMTS2; ADA
hsa-mir-29c	TRIM63; TMEM132A; SYPL2; SPARC; SMTNL2; SLC16A1; SFXN3; SERPINH1; RCC2; PXDN; PTHLH; PDGFRB; NID1; NFIX; MYBL2; MFAP2; LOXL2; ITGA6; HTR7; HAS3; FOS; EN1; EMP1; EHD2; DNMT3B; CSPG4; COL7A1; COL6A3; COL6A2; COL5A3; COL5A2; COL4A6; COL4A5; COL4A2; COL4A1; COL3A1; COL27A1; COL1A2; COL1A1; COL11A1; CD276; CA3; C1QTNF6; BMP1; AMOT; ADAMTS2; ADA
hsa-mir-301a	TTYH3; TNFSF10; TMOD1; SASH1; RRAGD; RASD1; RAB34; PMEPA1; NPNT; MYO10; MET; KLF7; INHBA; IMPDH1; GJA1; FZD6; FYCO1; FRZB; EGLN3; CEP55; ADAM12
hsa-mir-130b	TTYH3; TNFSF10; TMOD1; SASH1; RRAGD; RASD1; RAB34; PMEPA1; NPNT; NDRG2; MYO10; MET; KLF7; INHBA; IMPDH1; GJA1; FZD6; FYCO1; FRZB; EGLN3; CEP55; ADAM12
hsa-mir-454	TTYH3; TNFSF10; TMOD1; SULF1; SASH1; RRAGD; RASD1; RAB34; PXDN; PMEPA1; NPNT; MYO10; MET; KLF7; INHBA; IMPDH1; GJA1; FZD6; FRZB; EGLN3; CEP55; ADAM12
hsa-mir-31	UCN2; TFRC; SPARC; SLC2A4; SH3BGRL2; RAB31; PTGFRN; PRELP; PAX9; OAS2; EMP1; EGLN3; DCBLD2; CDK1; APP; AGRN
hsa-mir-133b	VEGFC; TTYH3; TRAM2; SLC7A8; SAMD5; NDRG1; MSN; MMP14;

	LDOC1; LAMB3; IFIT2; HLF; FSCN1; DUSP1; DSN1; DCBLD1; CTSV; CDCA8
hsa-mir-503	WNT7A; USP2; PTK7; KIF23; HMGA2; CXCL10; CDCA4; CCNE1; CAPN6; ANLN
hsa-mir-195	WNT7A; WIF1; TGFBR3; TFRC; SH2D2A; PTHLH; PDK4; KIF23; HMGA2; GNA12; FZD6; CXCL10; COL12A1; CEP55; CDCA4; CCNE1; CALU; BPIFA1; APP; ANLN; ALOX12
hsa-mir-424	WNT7A; WIF1; TGFBR3; TFRC; SH2D2A; PTHLH; PDK4; KIF23; HMGA2; GNA12; FZD6; CXCL10; COL12A1; CEP55; CDCA4; CCNE1; CAPN6; CALU; BPIFA1; APP; ANLN; ALOX12
hsa-mir-204	ZBTB7C; SPARC; SLC16A6; SAMD5; PRR11; NXPH4; MYO10; LMOD3; HMGA2; FJX1; COL5A3; ATP13A4
hsa-mir-183	ZIC2; TRDN; TPM2; THY1; SYPL2; PMEPA1; MSN; FHL1; FAT1; EXT1; COBL; CLIC4; CCNB1; ASPN
hsa-mir-96	ZIC2; TTYH3; SLC7A8; RCC2; PMEPA1; NETO2; MSN; FN1; FHL1; EXT1; DTL; COL4A6; COBL; BASP1; ASPN

Supplementary Table 2

Abbreviations and Full Names

Abbreviations	Full Names
OSCC	Oral Squamous Cell Carcinoma
circRNA	Circular RNA
ceRNA	Competing Endogenous RNA
GEO	Gene Expression Omnibus
PPI	Protein–Protein Interactions
HPV	Human Papillomavirus
miRNA	MicroRNA
DEcircRNAs	Differentially Expressed circRNAs
DEmiRNAs	Differentially Expressed miRNAs
DEmRNAs	Differentially Expressed mRNAs
NCBI	National Center for Biotechnology Information
GDC	Genomic Data Commons
KEGG	Kyoto Encyclopedia of Genes and Genomes
AIC	Akaike Information Criterion
ROC	Receiver Operating Characteristic
TIDE	Tumour Immune Dysfunction and Exclusion
ICB	Immune Checkpoint Blockade
CI	Concordance Index
CTL	Cytotoxic T Lymphocytes
MDSCs	Myeloid-derived Suppressor Cells
CAFs	Cancer-associated Fibroblasts
EMT	Epithelial-mesenchymal Transformation
UCSC	University of California， Santa Cruz
PiT1	Phosphate Transporter 1
PITX2	Paired-like Homeodomain Transcription Factor 2
PD-1	Programmed Cell Death Protein-1
TPI	Tumour Promoting Inflammation
